# Unraveling the role of RDM1 in head and neck squamous cell carcinoma: insights into DDR regulation and antioxidant response

**DOI:** 10.3389/fmolb.2026.1944888

**Published:** 2026-10-13

**Authors:** Fengpin Wang, Jie Wu, Wenjie Wang, Ruilin Zhao, Yunqi Mao, Fuhan Wu, Yixuan Li, Pingjiang Ge, Weiqun Zhu, Xiaodong Yan, Yanfang Zheng, Zhongming Lu

**Affiliations:** 1 Affiliated Cancer Hospital and Institute of Guangzhou Medical University, Guangzhou, China; 2 Department of Geriatrics, Guangdong Provincial Geriatrics Institute, Guangdong Provincial People’s Hospital, Guangdong Academy of Medical Sciences, Southern Medical University, Guangzhou, China; 3 Department of Otolaryngology-Head and Neck Surgery, Guangdong Provincial People’s Hospital, Guangdong Academy of Medical Sciences, Southern Medical University, Guangzhou, China; 4 Department of Otolaryngology Guangdong Provincial Key Laboratory of Intelligent Port Security Inspection, Guangzhou, Guangdong, China

**Keywords:** antioxidant response, DNA damage response, head and neck squamous cell carcinoma, KEAP1, Nrf2, RDM1

## Abstract

Advanced Head and Neck Squamous Cell Carcinomas (HNSCCs) pose a significant clinical challenge due to their poor survival rates. The DNA damage response (DDR) pathway plays a crucial role in the progression and treatment of advanced HNSCC, prompting the need to identify the underlying molecular mechanisms and develop innovative therapeutic strategies. One promising candidate is the RAD52 motif-containing protein 1 (RDM1), which functions as a regulator of DDR and exhibits context-dependent roles in tumorigenesis. However, the precise function and molecular mechanism of RDM1 in HNSCC remain poorly understood. In our study, we observed a relatively high expression of RDM1 in HNSCC tumor tissues. Functional experiments revealed that RDM1 contributed to increased cell proliferation and suppressed apoptosis in HNSCC cells, indicating its oncogenic role in HNSCC development. By modulating the levels of RAD52/TP53 and CDKN1A/CDK6 proteins, RDM1 played a critical role in regulating DDR and the cell cycle in HNSCC cells. Additionally, our investigations uncovered a novel role of RDM1 in the antioxidant response, wherein it facilitated the accumulation of NRF2 proteins. Interestingly, we found that RDM1 interacted with KEAP1 protein to promote NRF2 accumulation, ultimately promoting HNSCC progression. These findings establish the oncogenic role of RDM1 in HNSCC and emphasize the novel RDM1-KEAP1-NRF2 axis as a mechanism underlying the antioxidant response. They also highlight the potential of RDM1 as a therapeutic target for HNSCC, while its prognostic value warrants further validation in larger independent cohorts.

## Graphical Abstract

**Figure:**
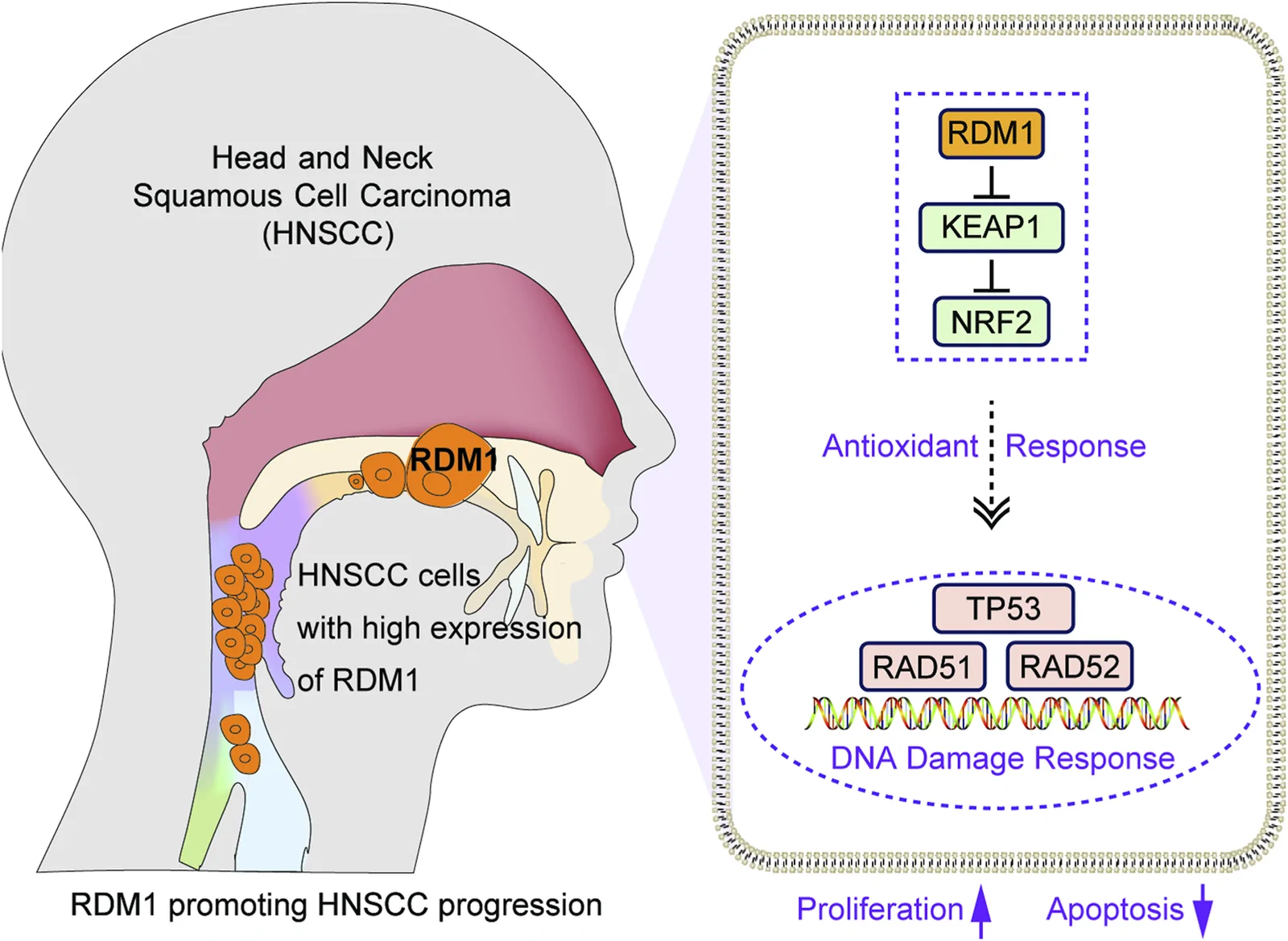

## 1 Introduction

Head and neck squamous cell carcinoma (HNSCC) is the sixth most common cancer worldwide, with the majority of aggressive cases being HPV-negative and associated with tobacco and alcohol use (Sung et al., 2021; Johnson et al., 2020; Mody et al., 2021; Chow, 2020). Despite therapeutic advances, approximately two-thirds of patients present with advanced-stage disease, and the long-term survival rate remains stagnant at around 50% (Van den Bossche et al., 2022). Dysregulation of the DNA damage response (DDR) is a hallmark of HNSCC progression, driven by frequent alterations in TP53, CDKN2A, CCND1, and CDK6 (Hammerman et al., 2015). Therefore, identifying novel therapeutic targets within DDR-related pathways is crucial for improving outcomes in advanced HNSCC.

The transcription factor NRF2 orchestrates the cellular antioxidant response and is negatively regulated by KEAP1, which targets NRF2 for ubiquitin–proteasome degradation (Yamamoto et al., 2018; Hayes et al., 2020; Sies and Jones, 2020). In HNSCC, NRF2 hyperactivation—often through KEAP1 mutation or altered upstream regulation—promotes tumor progression, chemo/radioresistance, and poor prognosis (Rojo de la Vega et al., 2018; Menegon et al., 2016; Kim et al., 2010). Disruption of KEAP1–NRF2 binding represents a key mechanism by which NRF2 accumulates and drives antioxidant gene expression.

RDM1 is a protein containing a RAD52 motif and an RNA recognition motif, implicated in DNA damage repair and cell-cycle regulation across multiple cancer types (Hamimes et al., 2005; Messaoudi et al., 2007; Tong et al., 2018; Chen et al., 2019; Tong et al., 2020; Li et al., 2017; Sheng et al., 2021; Xie et al., 2019; Chen et al., 2020). While RDM1 is predominantly cytoplasmic, its downstream effects on nuclear DDR proteins suggest cytoplasm-to-nucleus signaling (Messaoudi et al., 2007). However, the cytoplasmic mechanism of RDM1 and its potential link to antioxidant signaling remain unknown.

In this study, we demonstrate that RDM1 is highly expressed in HNSCC and promotes tumor progression through coordinated regulation of DDR-associated signaling and the KEAP1–NRF2 antioxidant axis. We show that RDM1 physically interacts with KEAP1, promotes NRF2 protein accumulation, and modulates RAD52/TP53 and CDKN1A/CDK6 pathways. These findings establish RDM1 as a potential therapeutic target in HNSCC.

## 2 Materials and methods

### 2.1 Clinical specimen and TCGA database

Human HNSCC samples and adjacent normal tissues were obtained from patients at the Department of Otolaryngology Head and Neck Surgery, Guangdong Provincial People’s Hospital. Written informed consent was obtained from all participants. This study was approved by the Research Ethical Committee of the Guangdong Provincial People’s Hospital and Guangdong Academy of Medical Sciences (Approval No. KY2020-521-01). In addition, clinical samples from TCGA were downloaded from the TCGA database (https://tcga-data.nci.nih.gov/) for and mRNA expression profiling. This study utilized the TCGA database to obtain the relative expression levels of RDM1 mRNA in patients with head and neck squamous cell carcinoma. Correlation analysis and Kaplan-Meier survival analysis were performed.

### 2.2 Cell culture

Human HNSCC cells (Hep2 and Fadu) were all obtained from the National Collection of Authenticated Cell Cultures, Shanghai, China. Cells were grown in Dulbecco’s modified eagle medium (DMEM) with 10% fetal bovine serum (FBS) and 1% penicillin-streptomycin at 37 °C in a humidified incubator with 5% CO2.

### 2.3 Cell-derived xenotransplantation (CDX)

To validate the role of the RDM1/NRF2 axis in the development of head and neck squamous cell carcinoma (HNSCC), we utilized the nude mouse xenograft model. Totally, 20 Female BALB/c nude mice (4–5 weeks old, average weight: 12 g) were purchased from Guangdong Experimental Animal Center and divided into the RDM1 KD group (n = 10) and the KD Ctrl group (n = 10). Human Fadu cells were micro-injected at a density of 4 × 10^6^ into the right upper limbs of these mice. Tumor sizes were measured on the 12th, 15th, 18th, 21st, 24th, 27th, and 30th days after transplantation. All animals were sacrificed on day 35 for final tumor collection and weighing. In the shRDM1 group, 2 out of 10 mice developed measurable tumors, while 8 mice did not develop palpable tumors during the observation period; all 10 mice in the shCtrl group developed tumors. For the tumor growth curve analysis, only animals with measurable tumors were included (n = 10 for shCtrl, n = 2 for shRDM1). For the final tumor weight analysis, all animals were included, with non-tumor-bearing animals assigned a tumor weight of 0 (n = 10 per group). For IHC quantification, all tumor-bearing animals were selected for staining and scoring. Tumor size was calculated as V = L × W × W/2 (L: major axis; W: minor axis). For immunohistochemical staining, each tissue sample was paraffin-embedded and fixed in 4% formalin immediately. All animal care procedures and experiments received approval from the Research Ethical Committee of the Guangdong Provincial People’s Hospital and Guangdong Academy of Medical Sciences (Approval No. KY2020-521-01).

### 2.4 RT-qPCR assay

We used reverse transcription-quantitative polymerase chain reaction (RT-qPCR) to validate the expression levels of RDM1 mRNA in tumor tissue and adjacent normal tissue and HNSCC cells. Total RNA was extracted from HNSCC cells using Trizol agent (Invitrogen). cDNA synthesis was performed according to the manufacturer’s instructions (TaKaRa), and RT-qPCR was performed with SYBR Premix Ex Taq II (TaKaRa) using a LightCycler system (Roche). The RDM1 were calculated using the 2^−ΔΔCT^ method, normalized to GAPDH. The RT-qPCR primers target a conserved region present in all major RDM1 isoforms. The primers were: RDM1 forward: 5′- GCCCATCCTGGTTTCTATGCC-3′, and reverse: 5′- AGACGAACCTTGACTGGAGAT-3’; GAPDH forward: 5′-TGACTTCAACAGCGACACCCA-3′, and reverse: 5′-CACCCTGTTGCTGTAGCCAAA-3’.

### 2.5 Western blot assay

To verify the expression of RDM1 protein and its effects after overexpression and knockdown, we employed the Western blot technique. Cells were lysed in RIPA buffer with protease inhibitor cocktail on ice for 30 min. Cell lysates were centrifuged at 12,000r/min for 15 min at 4 °C and supernatants were collected. Protein concentration was determined, and equal amounts were separated by sodium dodecyl sulfate polyacrylamide gel electrophoresis (SDS-PAGE), transferred to polyvinylidene fluoride (PVDF) membranes, and blocked in 5% skim milk for 2 hours. The PVDF membranes were then incubated overnight with appropriate dilutions of primary antibodies at 4 °C. After washing five times with TBST, the blots were incubated with HRP-conjugated secondary antibodies. Protein bands were visualized with SuperECL (Biosharp, China).

Primary antibodies used in this study were: human RDM1 (1:5000, Sigma: HPA024708; 1:2000; raised against the N-terminal region of the canonical RDM1 isoform, UniProt Q9H0B6-1), RAD52 (1:5000, Abcam: ab124971), TP53 (1:5000, Abcam: ab26), CDKN1A (1:2000, Abcam: ab109520), CDK6 (1:3000, CST: #3136), EZH2 (1:2000, Abcam: ab191080), HSP90AA1 (1:3000, CST: #4877), HSPA1A (1:3000, CST: #4873), NRF2 (1:2000, Abcam: ab31163), BCL2 (1:2000, Abcam: ab692), KEAP1 (1:2000, Abcam: ab227828), Flag (1:3000, Sigma: F1804) and GAPDH (1:5000, Santa-Cruz: sc-32233).

### 2.6 Cell transfection

To knock down RDM1 expression, specific small interfering RNAs (siRNAs) of RDM1 (siR#1: GGCCCATCCTGGTTTCTAT; siR#2: AGAAATCTCCAGTCAAGGT), along with control siRNA (NC) were designed and synthesized (GeneCloudBio, China). HNSCC cells were transfected with these siRNAs utilizing Lipofectamine 3000 (Invitrogen, United States). Knockdown efficiency was validated 24 h post-transfection through RT-qPCR and western blot assays. For stable knockdown, lentiviral vectors containing the target siRNAs were also synthesized (GeneCloudBio, China). Cells were infected with these lentiviruses in the presence of 1 μL Polybrene (5 μg/μL). After 72 h, puromycin selection (5 μg/mL) was initiated. Cells that became puromycin-resistant after 10 days were deemed stably transfected. To stably overexpress RDM1, a lentiviral vector carrying the full-length canonical RDM1 isoform (UniProt Q9H0B6-1, 335 amino acids, containing the RAD52 motif and RNA recognition motif, predominantly cytoplasmic) was designed and synthesized (GeneCloudBio, China). The transduction and selection procedures were the same as those for stable knockdown. Either knockdown or overexpression efficiency was confirmed via RT-qPCR and western blot assays.

### 2.7 Cell proliferation assay

To evaluate and compare the impact of RDM1 knockdown and overexpression on cell proliferation, we utilized the MTT cell viability assay and Celligo cell proliferation analysis. For MTT assay, Hep2 and FaDu cells infected with shRDM1 or siRNA Ctrl were seeded into a 96-well plate at a density of 1000 cells in 100 μL DMEM at 37 °C for 1, 2, 3, 4 or 5 days, followed by 10 μL of MTT reagent and incubated for 1 h. OD 450 nm was measured, and cellular proliferation curves were constructed using GraphPad Prism software. For Celligo assay, HNSCC cells are seeded into a 96-well plate at 37 °C for 1, 2, 3, 4 or 5 days, followed by counting using the image cytometer in EGFR field images.

### 2.8 Cell apoptosis assay

To evaluate and compare the effects of RDM1 knockdown and overexpression on cell apoptosis, we used the Annexin V-APC cell apoptosis assay and caspase-3/7 activity assay. For Annexin V-APC cell apoptosis assay, cells were harvested by centrifuging at 1500 rpm for 5 min and then washed with 1 × binding buffer and 1× staining buffer. About 5 μL of Annexin V-APC staining solution was added to 100 μL of cell suspension in the dark, followed by detection via flow cytometry. For caspase-3/7 activity assay, Caspase-Glo™ 3/7 Reagent was added directly to the HNSCC cells in 96-well plates and incubated for 1 h before recording luminescence.

### 2.9 Cell cycle assay

To detect the cell cycle of RDM1 knockdown and overexpression cells, we employed PI staining and flow cytometry. Cells cultured in 60-mm dishes were harvested, washed with PBS and fixed in 70% ethanol overnight at 4 °C. After centrifugation, the supernatants were discarded and cells were resuspended and incubated with PI at 37 °C for 30 min. Cell cycle was determined by flow cytometry (BD Accuri C6, San Diego, CA, United States). Data were analyzed by FlowJo V10 software (Tristar, CA, United States).

### 2.10 DCFDA/H2DCFDA - cellular ROS assay

To evaluate and compare the antioxidant response of RDM1 knockdown and overexpression cells, we utilized the DCFDA/H2DCFDA ROS assay (Abcam: ab113851). Cells were collected cells in tubes, followed by staining with DCFDA for 30 min and detecting with flow cytometer.

### 2.11 Transcriptome profiling

To analyze differentially expressed genes regulated by RDM1, we performed Affymetrix microarray transcriptome profiling. Total RNA was extracted from HNSCC cells using TRIzol (Invitrogen). RNA quality was validated with a NanoDrop spectrophotometer and an Agilent 2100 bioanalyzer. Biotinylated cRNA was synthesized, fragmented, and hybridized to Affymetrix PrimeView Human Gene Expression Arrays. Array staining and scanning were performed according to the manufacturer’s protocol. Raw CEL files were normalized using the RMA algorithm implemented in the R `affy` package. Differentially expressed genes were defined using thresholds of |log_2_ fold change| > 1 and adjusted *P* < 0.05. Normalized expression data were subjected to principal component analysis, volcano plot visualization, and functional enrichment analyses (KEGG and Ingenuity pathway analysis).

### 2.12 Immunoprecipitation and mass spectrometry detection

To detect the RDM1 interacting proteins, we used immunoprecipitation and mass spectrometry techniques. Affinity enrichment of Flag-Tag protein experiment was performed from RDM1-Flag overexpression Fadu cells. Cell cultured in a 10 cm dish were harvested. A total of 500 µg of cell lysis protein was added to spin columns (Pierce) containing anti-FLAG M2 affinity resin (Merck) and incubated rotating for 2 hours at 4 °C. Proteins were eluted with 50 μL of 100 μg/mL of 3X FLAG tag peptide (Merck) in solubilization buffer followed by acetone precipitation. Proteins were then pelleted for Mass Spectrometry detection. Immunoprecipitation enriches target proteins and their interacting proteins, while mass spectrometry identifies and quantifies the precipitated RDM1 proteins and their interacting partners.

### 2.13 Human proteome microarray assay

Recombinant RDM1 were expressed in 293F using the pCDNA3 vector expression system and purified by nickel chelating, ion exchange and gel filtration columns (GeneCloudBiotech, China). The recombinant RDM1-His fusion proteins were labeled with Cy5 dye for the Human Protein Microarray (CDI Laboratories, United States). The RDM1-Cy5 protein was diluted to 0.01 mg/mL in blocking buffer and incubated on the blocked proteome microarray at room temperature for 1 h, followed by scanning with a GenePix 4000B (Axon Instruments, United States) to visualize and record the results. To generate the candidate list of RDM1 interacting proteins, the signal-to-noise ratios (SNR = (F635 mean–B635 mean)/B635 s.d.) were calculated for all the spots.

### 2.14 Immunohistochemistry assay

To detect the expression levels of RDM1 and NRF2 in tumor tissue, we employed Immunohistochemistry assay. Paraffin-embedded sections of HNSCC tissues were deparaffinized and rehydrated through xylene and graded ethanol series, followed by IHC staining to evaluate the protein expression using antibodies against RDM1 and NRF2 at 4 °C overnight. Subsequently, the tissues were incubated with a secondary antibody (Servicebio, China) for 1 h at room temperature. Finally, the tissue sections were mounted using neutral resin and visualized under a fluorescence microscope (Olympus, Japan). Staining scores were assessed based on both area coverage and staining intensity. The percentage of the stained area was scored as follows: 0 for <5%, 1 for 5%–25%, 2 for 25%–50%, 3 for 50%–75%, and 4 for >75%. Staining intensity was categorized as 0 for no staining, 1 for weak staining, 2 for moderate staining, and 3 for intense staining. The overall staining score was independently evaluated by two pathologists and calculated as the sum of the area and intensity scores.

### 2.15 Statistical analysis

The results are represented as mean ± SD. All *in vitro* experiments were performed with at least three independent biological replicates (n = 3) unless otherwise stated; *in vivo* experiments included 10 animals per group (n = 10). Statistically significant differences between two groups were determined by two-sided Student’s t-test, and comparisons among multiple groups were performed using one-way ANOVA followed by Tukey’s *post hoc* test. For cell growth and xenograft growth curve analyses, repeated-measures two-way ANOVA was used. The Kaplan-Meier method and log-rank test were used to evaluate overall survival (OS). Normality was assumed based on previous experience with these assays. A P value <0.05 was considered statistically significant (*P < 0.05, **P < 0.01). All statistical analyses were performed using GraphPad Prism 8.1 (GraphPad Software, United States).

### 2.16 Co-immunoprecipitation assay

To validate the protein-protein interaction between RDM1 and KEAP1, co-immunoprecipitation (Co-IP) was performed. HNSCC cells stably expressing Flag-tagged RDM1 were lysed in NP-40 lysis buffer (50 mM Tris-HCl pH 7.4, 150 mM NaCl, 1% NP-40, 1 mM EDTA) supplemented with protease inhibitor cocktail (Roche). Cell lysates were pre-cleared with protein A/G agarose beads for 1 h at 4 °C, then incubated with anti-Flag M2 affinity resin (Sigma, A2220) overnight at 4 °C with gentle rotation. The resin was washed five times with lysis buffer, and bound proteins were eluted by boiling in 2 × SDS loading buffer. Eluted proteins were separated by SDS-PAGE and subjected to western blot analysis using antibodies against Flag, KEAP1, and RAD52.

## 3 Results

### 3.1 Elevated RDM1 expression in HNSCC and its association with prognosis

To evaluate the clinical relevance of RDM1 in HNSCC, we conducted a comprehensive analysis of RDM1 expression across three independent HNSCC cohorts. Initially, we examined the expression of RDM1 in HNSCC tumors and matched normal tissues using the TCGA database. Our analysis revealed a significantly higher expression of RDM1 in HNSCC tumor tissues compared to normal tissues (Supplementary Figure S1; Figure 1A). To validate these findings, we further investigated the relative expression of RDM1 in tumor and adjacent normal tissues from HNSCC patients treated at local hospitals. Using RT-qPCR, we confirmed that RDM1 was highly expressed in HNSCC tumor tissues compared to adjacent normal tissues (Figure 1B). Moreover, we analyzed the expression data of HNSCC patients from a separate study to determine the RDM1 expression patterns (Huang et al., 2021). Consistently, our analysis demonstrated a significantly elevated expression level of RDM1 in HNSCC tumor tissues compared to normal tissues (Figure 1C). Finally, we examined the association between RDM1 expression and the prognosis of HNSCC patients using survival data from the TCGA dataset. Kaplan–Meier survival analysis revealed a trend toward poorer overall survival in HNSCC patients with higher RDM1 expression; however, this association did not reach statistical significance and should be interpreted cautiously (Figure 1D). Taken together, our analyses consistently demonstrate a heightened expression of RDM1 in HNSCC patients, suggesting its potential involvement in HNSCC oncogenesis.

**FIGURE 1:**
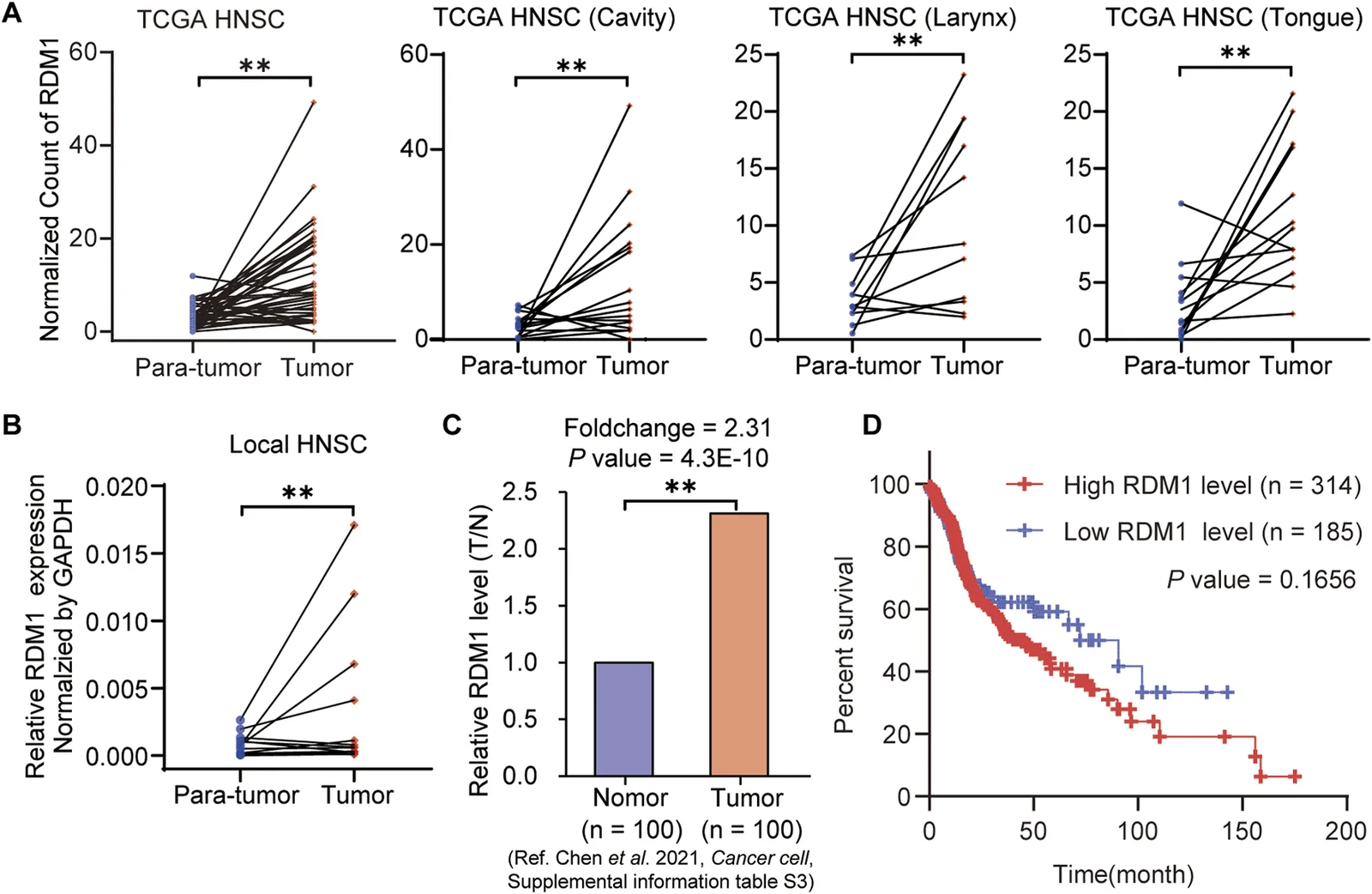
Association of highly expressed RDM1 in HNSCC tissues. (**A,B)** Analysis of RDM1 mRNA expression in tumor and adjacent normal tissues of HNSCC patients from both TCGA datasets and our local hospital was conducted. **(C)** RDM1 mRNA expression levels were assessed in the HNSCC cohort (reference (Huang et al., 2021; Supplementary Table S1). **(D)** Kaplan-Meier survival analysis was performed to evaluate the impact of RDM1 expression on overall survival in patients with HNSCC, using TCGA datasets. **P value <0.01.

### 3.2 Impact of RDM1 on cell proliferation and apoptosis in hnscc cells

To investigate the oncogenic role of RDM1 in head and neck squamous cell carcinoma (HNSCC), we first assessed the expression level of RDM1 in HNSCC cell lines using reverse transcription-quantitative polymerase chain reaction (RT-qPCR). Our results showed that the expression of RDM1 was higher in Fadu cells compared to Hep2 cells (Figure 2A). FaDu cells were selected for subsequent mechanistic experiments because they derive from hypopharyngeal SCC, an aggressive HNSCC subtype with poor prognosis; they express substantially higher endogenous RDM1 than Hep2 cells, providing a physiologically relevant model for knockdown studies; and they showed high transfection efficiency and robust phenotypic responses. Key findings were independently validated in Hep2 cells (Supplementary Figure S2) to ensure generalizability. To manipulate the expression of RDM1, we designed two short hairpin RNAs (shRNAs) and constructed lentiviruses to establish stable RDM1 knockdown cells. Additionally, RDM1 overexpression cells were generated using lentiviruses carrying an RDM1 overexpression construct. We confirmed the knockdown and overexpression efficiency of RDM1 through RT-qPCR and Western blot assays, which showed significant alterations in both RDM1 mRNA and protein levels in the respective cells (Figures 2B,C). To assess the impact of RDM1 on cell proliferation, we performed an MTT assay. Our results demonstrated that RDM1 knockdown significantly inhibited cell viability, while RDM1 overexpression enhanced it (Figures 2D,E). Furthermore, we conducted a celligo assay to evaluate cell proliferation and found that RDM1 knockdown suppressed cell proliferation while overexpression promoted it in the tumor cells (Figures 2F,G). To investigate the effect of RDM1 on apoptosis, we utilized Annexin V staining and flow cytometry. Our findings revealed that RDM1 knockdown led to a significant increase in apoptosis, while overexpression resulted in a decrease in apoptosis (Figures 2H,I). To consolidate our findings, we also performed siRNA transfection experiments in Hep2 cells. Similar to our findings in Fadu cells, we observed that RDM1 knockdown significantly inhibited cell activity and increased apoptosis levels in Hep2 cells (Supplementary Figure S2). Collectively, our experiments indicate that RDM1 promotes proliferation and imparts resistance to apoptosis in HNSCC.

**FIGURE 2:**
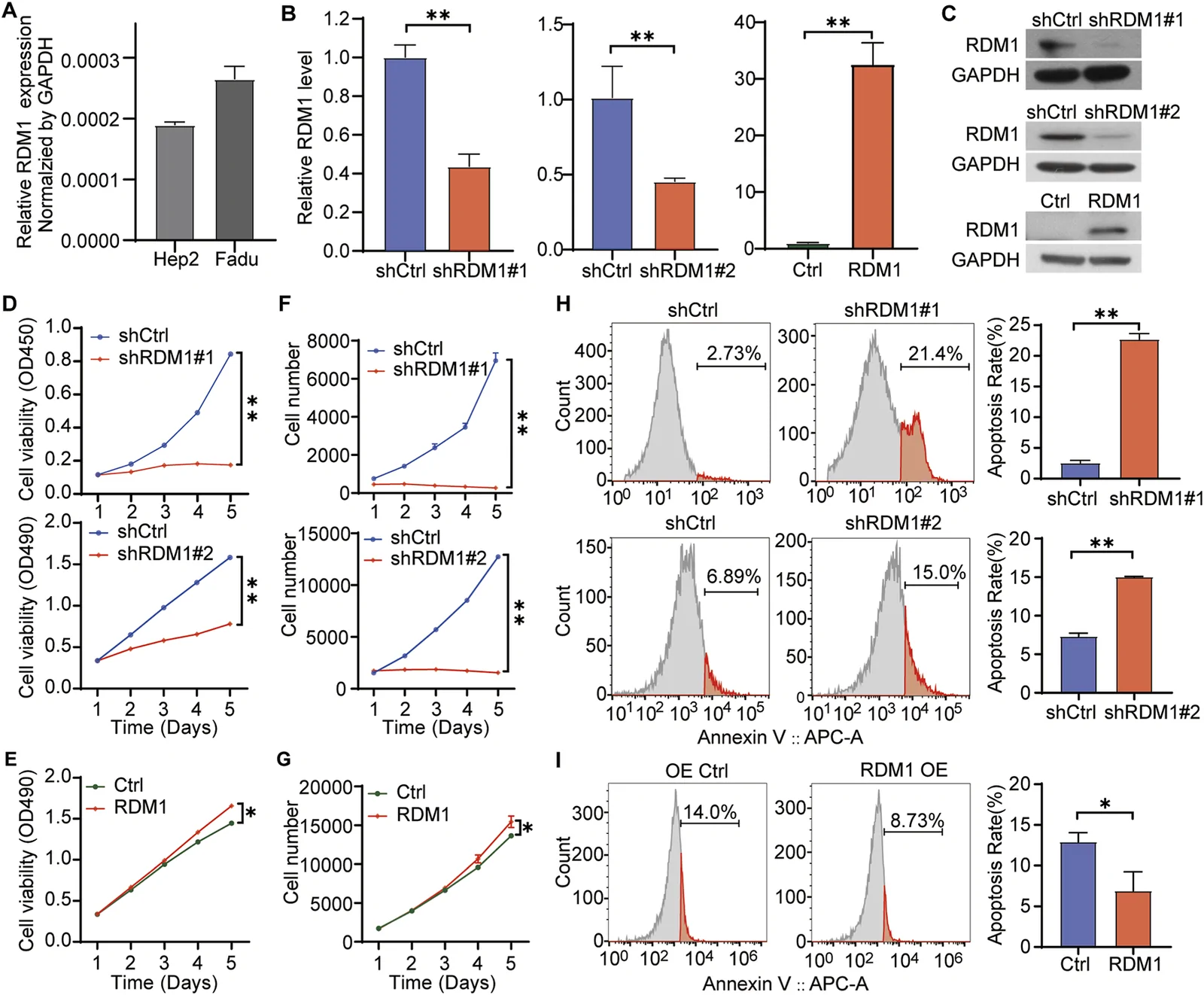
Highly expressed RDM1 promotes cell proliferation and suppresses apoptosis in HNSCC cells. **(A)** The relative expression of RDM1 was assessed in two HNSCC cell lines. **(B,C)** The efficiency of RDM1 knockdown (shRDM1) and overexpression was confirmed using RT-qPCR and western blot analysis. **(D,E)** The cell viability of RDM1 knockdown and overexpression cells was evaluated using the MTT assay. **(F,G)** Cell proliferation in RDM1 knockdown and overexpression cells was assessed using the Celligo assay. **(H,I)** Cell apoptosis in RDM1 knockdown and overexpression cells was measured using the Annexin V-APC apoptosis assay. *P value <0.05, **P value <0.01.

### 3.3 RDM1’s role in DDR and cell cycle regulation in HNSCC cells

To investigate the molecular mechanism of RDM1, we performed Transcriptome profiling by Affymetrix microarray using RDM1 knockdown cell lines. Subsequently, we conducted Principal Component Analysis (PCA) and Volcano map analysis to identify differentially expressed genes between the RDM1 knockdown cells and the control (Figure 3A). The Ingenuity Pathway Analysis (IPA) database was utilized to investigate the enriched canonical pathways of these genes. The results indicated that DDR and cell cycle regulation pathways were highly enriched (Figure 3B).

**FIGURE 3:**
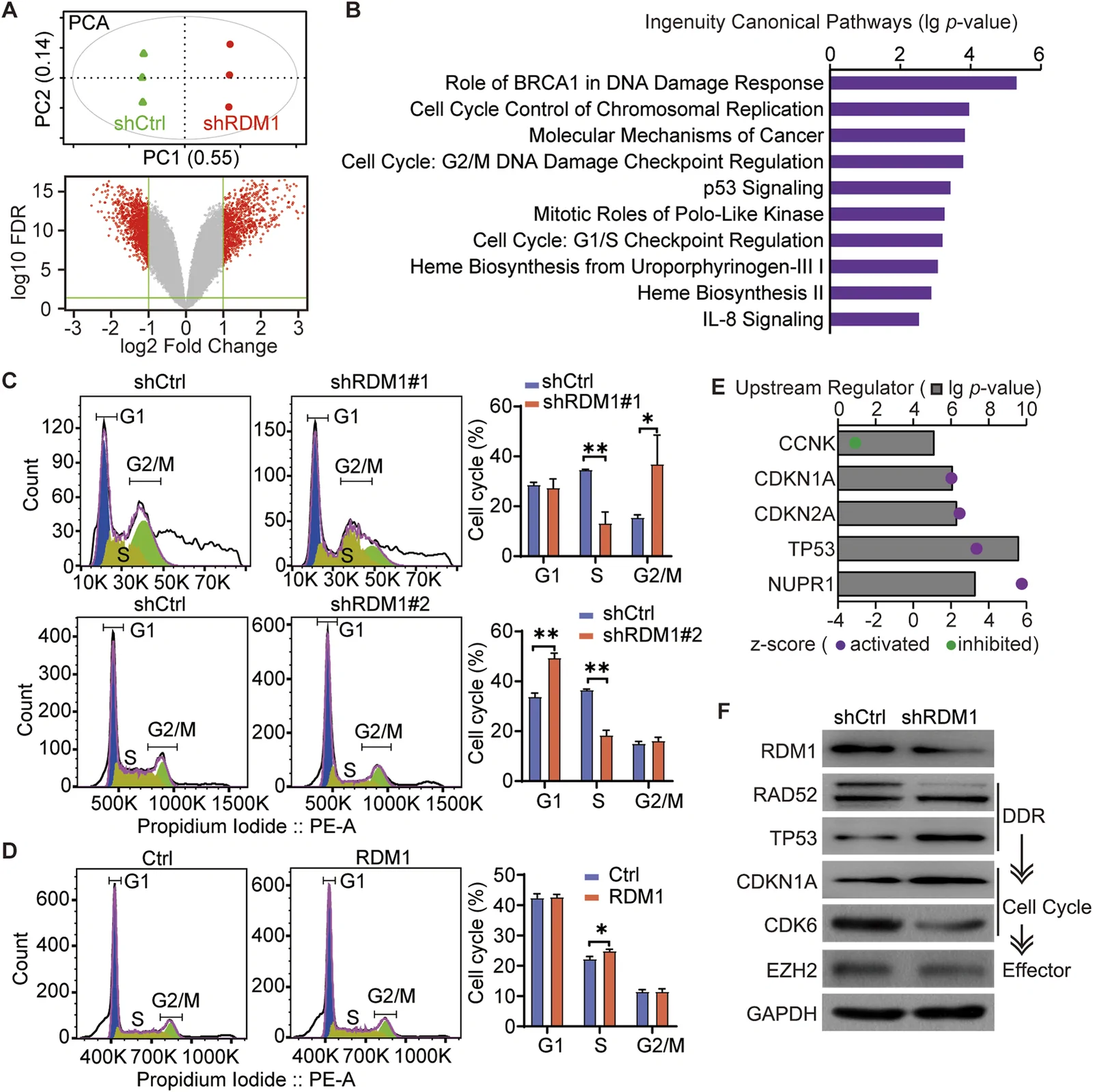
RDM1 regulates DDR and cell cycle through the RAD52/TP53 and CDKN1A/CDK6 axis in HNSCC cells. **(A)** Principal Component Analysis (PCA) and Volcano map illustrating the differentially expressed genes regulated by RDM1, as determined by Transcriptome profiling by Affymetrix microarray. **(B)** Ingenuity Pathway Analysis (IPA) was used to analyze the differentially expressed genes and identify potential pathways involved in RDM1 regulation. **(C,D)** PI-based cell cycle assay was performed to assess the impact of RDM1 knockdown and overexpression on cell cycle progression in HNSCC cells. **(E)** IPA was employed to identify the upstream regulators of the differentially expressed genes, revealing potential molecular factors influencing the observed gene expression changes. **(F)** Western blot analysis was conducted to examine the protein levels of key players involved in DNA damage response (DDR) and cell cycle regulation. *P value <0.05, **P value <0.01.

To further understand the involvement of RDM1 in the DDR and cell cycle, we performed PI staining and flow cytometry experiments. Our findings demonstrated that knocking down RDM1 resulted in a decrease in the proportion of cells in the S phase, while overexpression of RDM1 increased it (Figures 3C,D). These results suggest that RDM1 plays a role in regulating DNA during the S phase of the cell cycle in HNSCC.

Moreover, through the prediction of upstream regulators of the differentially expressed genes, we identified significant activation of TP53 and CDKN1A/2A signaling (Figure 3E). DDR is known to block the cell cycle and promote DNA repair (Hernández Borrero and El-Deiry, 2021). To investigate this, we conducted a western blot assay to detect proteins involved in DDR and cell cycle regulation, including RAD52, TP53, CDKN1A, CDK6, and EZH2 (Hernández Borrero and El-Deiry, 2021; Knudsen et al., 2019; Müller et al., 2020). Our findings demonstrated that knocking down RDM1 led to a significant decrease in RAD52 protein level, but an increase in TP53 protein level (Figure 3F). This suggests that RDM1 modulates DDR-associated signaling pathways in HNSCC cells. Additionally, RDM1 knockdown was found to increase CDKN1A protein level while decreasing CDK6 protein level (Figure 3F), indicating that RDM1 is involved in DNA synthesis during the S phase of the cell cycle. Furthermore, the downstream effector EZH2 protein was suppressed in RDM1 knockdown cells (Figure 3F). These findings suggest that RDM1 is associated with DDR-related signaling through modulation of RAD52/TP53 protein levels and regulates the cell cycle through the CDKN1A/CDK6 pathway.

### 3.4 RDM1’s involvement in ER stress, antioxidant response, and NRF2/BCL2 signaling in HNSCC

RDM1 is predominantly localized in the cytoplasm, necessitating cytoplasm-to-nucleus signaling for its impact on the DNA damage response (DDR) and cell cycle regulation. In order to elucidate the molecular mechanism underlying RDM1’s regulation of these processes, we conducted a KEGG analysis using transcriptome data. The analysis revealed that HPV infection, MAPK signaling, protein processing in the endoplasmic reticulum (ER), and cell cycle were the major pathways enriched in our study (Figure 4A). Previous research has demonstrated that RDM1 activates MEK/ERK signaling (Sheng et al., 2021). Additionally, our attention was drawn to protein processing in the ER, which is involved in ER stress (Chen and Cubillos-Ruiz, 2021). To investigate this further, we assessed the expression of molecular chaperones associated with ER stress using both RT-qPCR and western blot assays. Our findings indicate that knockdown of RDM1 significantly increases the expression of chaperones (Figures 4B,C), suggesting a potential role for RDM1 in the ER stress response.

**FIGURE 4:**
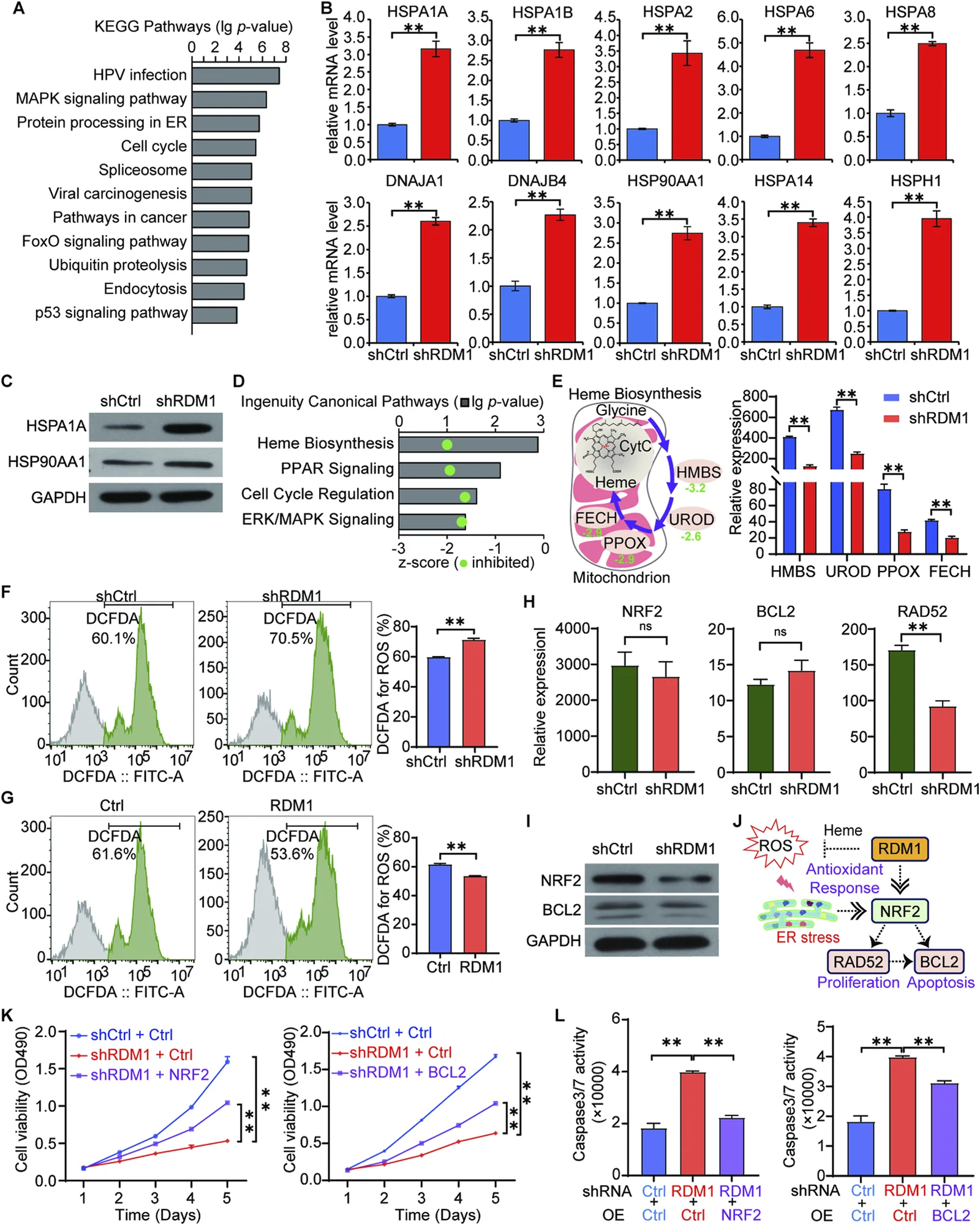
RDM1 promotes antioxidant resistance and is involved in apoptosis of HNSCC cells. **(A)** KEGG analysis was performed to identify differentially expressed genes. **(B)** The relative expression levels of chaperone genes involved in the MAPK signaling pathway and protein processing in the endoplasmic reticulum (ER) were measured using RT-qPCR. **(C)** Western blot analysis was conducted to assess the expression of the chaperones HSP1A1 and HSP90AA1. **(D)** Ingenuity pathway analysis (IPA) revealed an inhibition of the canonical pathway. **(E)** The heme biosynthesis canonical pathway was significantly inhibited and the corresponding genes were downregulated. **(F,G)** DCFDA ROS assay was performed to evaluate the antioxidant response in RDM1 knockdown and overexpression cells. **(H)** The relative expression levels of NRF2, RAD52, and BCL2 were determined in RDM1 knockdown cells based on transcriptome analysis. **(I)** Western blot analysis was employed to measure the protein levels of NRF2 and BCL2 in RDM1 knockdown cells. **(J)** A schematic diagram depicting RDM1 as a common upstream regulator of the NRF2/antioxidant and RAD52/DDR pathways. **(K)** MTT assay was conducted to assess cell viability in the context of proliferation rescue by NRF2 and BCL2 overexpression, respectively. **(L)** Caspase-3/7 activity assay was performed to evaluate cell apoptosis in the context of functional rescue by NRF2 and BCL2 overexpression, respectively. **P value <0.01.

We conducted a comprehensive analysis to further investigate the significantly inhibited canonical pathways using the IPA database. Our findings indicated that the heme biosynthesis pathway was significantly inhibited in cells with RDM1 knockdown (Figure 4D). Additionally, the gene expression related to heme biosynthesis was significantly decreased in these cells (Figure 4E). Heme, an essential component of the cytochrome C protein, plays a crucial role in oxidative phosphorylation within mitochondria (Alvarez-Paggi et al., 2017). The observed inhibition of the heme biosynthesis pathway in RDM1 knockdown cells suggests that RDM1 is involved in oxidative phosphorylation. Overall, our results provide evidence that RDM1 is associated with ER stress and oxidative phosphorylation, suggesting that RDM1 may play a role in antioxidant response. To further examine the role of RDM1 in antioxidant response, we measured the levels of reactive oxygen species (ROS) in cells with RDM1 knockdown and overexpression. Our results showed that RDM1 knockdown led to an increase in ROS levels, while RDM1 overexpression inhibited ROS production (Figures 4F,G). These findings highlight the critical role of RDM1 in antioxidant response. To gain insight into the underlying mechanisms, we investigated the expression levels of NRF2 and effector BCL2 mRNA in transcriptomic datasets and performed western blot assays to assess their protein levels in RDM1 knockdown cells. Interestingly, while RDM1 knockdown significantly inhibited the protein levels of NRF2 and BCL2, there was no notable change in their mRNA levels (Figures 4H–J), indicating post-translational regulation (protein stabilization) rather than transcriptional activation. Previous studies have demonstrated that NRF2 regulates the expression of RAD51 (Peng et al., 2021; Wang et al., 2019; Jayakumar et al., 2015). Moreover, our analysis of transcriptome data revealed that RAD52 mRNA levels were also decreased in RDM1 knockdown cells (Figure 4H), suggesting that NRF2 may be involved in RDM1-mediated transcriptional regulation of RAD52 (Figure 4J), although this link warrants further validation. Re-analysis of the transcriptome data further showed that several canonical NRF2 target genes, including BRCA1, OGG1, MUTYH, and MSH6, exhibited decreased mRNA expression upon RDM1 knockdown (Supplementary Table S2), consistent with reduced NRF2 transcriptional activity. Further research has shown that the RAD52/TP53 axis modulates the function of BCL2 (Hernández Borrero and El-Deiry, 2021; Nechiporuk et al., 2019), and BCL2 is a critical regulator of apoptosis (Gross and Katz, 2017). These findings suggest that NRF2/BCL2 signaling is essential for the functional role of RDM1. To validate the contribution of the NRF2/BCL2 axis in HNSCC progression, we conducted functional rescue experiments using MTT assays and caspase-3/7 activity assays. These experiments revealed that NRF2 and BCL2 significantly rescued cell viability and reduced apoptosis levels in RDM1 knockdown cells and NRF2/BCL2 overexpression cells compared to the RDM1 knockdown control (Figures 4K,L). Our findings provide further support for the assertion that RDM1 promotes HNSCC progression by promoting NRF2 protein accumulation and is associated with altered expression of NRF2 target genes and antioxidant function.

### 3.5 Elucidating RDM1’s role in NRF2 signaling and interaction with KEAP1 in HNSCC

To gain insights into the molecular mechanism underlying the enhancement of NRF2 signaling by RDM1, a comprehensive screening of the interacting proteins of RDM1 was conducted. This involved the utilization of immunoprecipitation and mass spectrometric detection (IP-MS) assay, as well as Human proteome microarray technology (Figures 5A,B). By integrating these two datasets, 1070 candidate proteins were identified for further analysis through KEGG pathways (Figure 5C). Notably, the top enriched pathways among these candidates were ribosome, proteasome, and protein processing in the ER. From the pool of candidates, 278 proteins were successfully detected through both IP-MS and protein microarray techniques. To narrow down the focus, we employed Uniprot and IPA databases to examine the proteins associated with mitochondria and/or tumor proliferation. This analysis resulted in the identification of 57 key candidate proteins (Supplementary Table S3). To illustrate the relationships among these proteins and their downstream effectors of RDM1, a protein-protein interaction (PPI) network analysis was performed. The findings highlighted two main clusters: the mitochondria/ROS cluster and the DDR/cell cycle cluster (Figure 5D).

**FIGURE 5:**
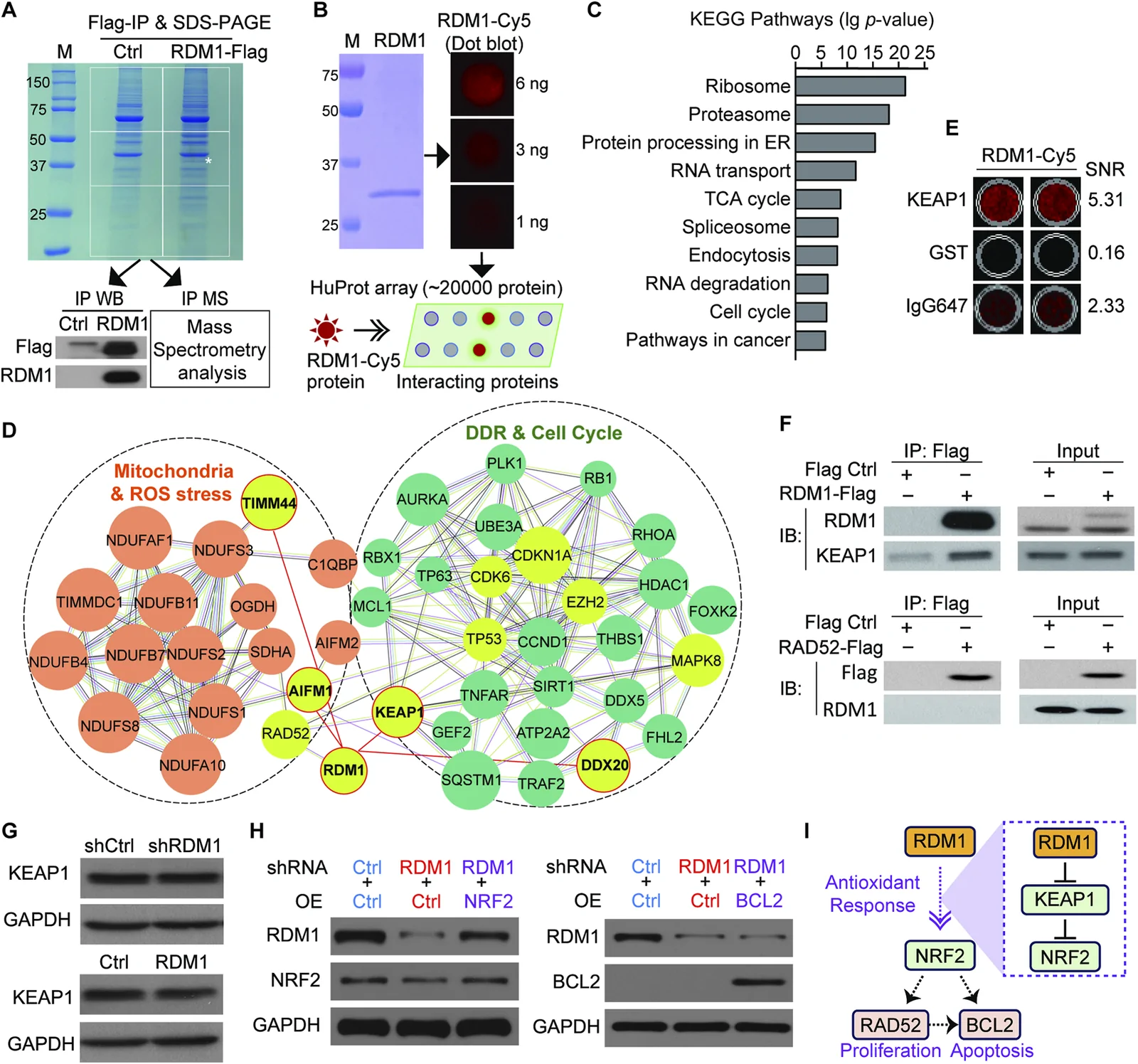
RDM1 interacts with KEAP1 and enhances NRF2 signaling to promote HNSCC progression. **(A)** Immunoprecipitation using anti-Flag beads, followed by mass spectrometry detection (IP-Mass), was performed in RDM1-Flag overexpression cells to identify RDM1 interacting proteins. **(B)** RDM1 recombinant protein was used to hybridize a HuProt proteome microarray (HPM) to identify potential interacting partners. **(C)** KEGG analysis was performed on the integrated data from IP-Mass and HPM datasets to gain insights into the pathways associated with RDM1 interacting candidates. **(D)** Protein-protein interaction (PPI) network analysis was conducted to reveal the relationships between RDM1 and its interacting candidates. **(E)** KEAP1 were found to be involved in the antioxidant response based on their high signal-noise ratio (SNR) levels. **(F)** Immuno-precipitation experiments using Flag antibody beads demonstrated that RDM1 interacted with KEAP1 but not RAD52 protein in RDM1-Flag overexpression cells. **(G)** Western blot analysis showed that KEAP1 protein levels remain unchanged upon RDM1 knockdown or overexpression. **(H)** Western blot analysis confirmed that RDM1 protein levels could be rescued by NRF2 overexpression but not by BCL2 overexpression. **(I)** A schematic diagram illustrating the RDM1-KEAP1-NRF2 signaling pathway involved in HNSCC progression.

We became interested in the KEAP1 protein, which is related to antioxidant response. By conducting a proteome microarray analysis, we observed that KEAP1 exhibited the highest signal-to-noise ratio (SNR) among the candidate proteins (Figure 5E). To validate the interaction between RDM1 and KEAP1, we performed a CoIP assay. The results confirmed the presence of the RDM1/KEAP1 complex (Figure 5F). Additionally, since RDM1 contains a RAD52-containing motif and can translocate to the nucleus under stress conditions, we investigated its interaction with RAD52. However, our findings indicated that RDM1 did not interact with RAD52 (Figure 5F). Thus, we concluded that RDM1 primarily interacts with KEAP1 rather than RAD52. Given RDM1’s ability to promote NRF2 protein accumulation, we hypothesized that RDM1 mainly functions by competing with NRF2 for binding to KEAP1, thereby preventing NRF2 degradation. To test this hypothesis, we examined the expression of KEAP1 in RDM1 knockdown and overexpression cells using western blot analysis. Our results showed that neither knockdown nor overexpression of RDM1 affected the levels of KEAP1 protein (Figure 5G). Furthermore, through a protein rescue experiment, we demonstrated that NRF2 overexpression could reverse the promotion of RDM1 protein accumulation, while BCL2 did not have the same effect (Figure 5H). These findings are consistent with a model in which RDM1 binds KEAP1 and interferes with KEAP1-mediated NRF2 degradation, although direct competitive binding remains to be definitively demonstrated, leading to the accumulation of NRF2 protein and thereby facilitating the progression of HNSCC (Figure 5I).

### 3.6 *In vivo* validation of RDM1’s tumor-promoting role and its association with NRF2

To further validate the role and function of RDM1/NRF2, we established a CDX model to investigate the cancer-promoting function of RDM1 and the regulatory mechanism of RDM1/NRF2. Our findings demonstrated that knockdown of RDM1 expression notably suppressed the growth of xenograft tumors (Figures 6A–C), but had no impact on the growth of nude mice (Figure 6D). Immunohistochemistry was employed to evaluate the expression level of NRF2 in the tumor, revealing a significant decrease in NRF2 protein levels in the RDM1 knockdown group (Figure 6E). These experiments demonstrate that RDM1 knockdown suppresses xenograft tumor growth *in vivo* and is associated with reduced NRF2 protein expression, supporting an *in vivo* link between RDM1 and NRF2 (Figure 6F).

**FIGURE 6:**
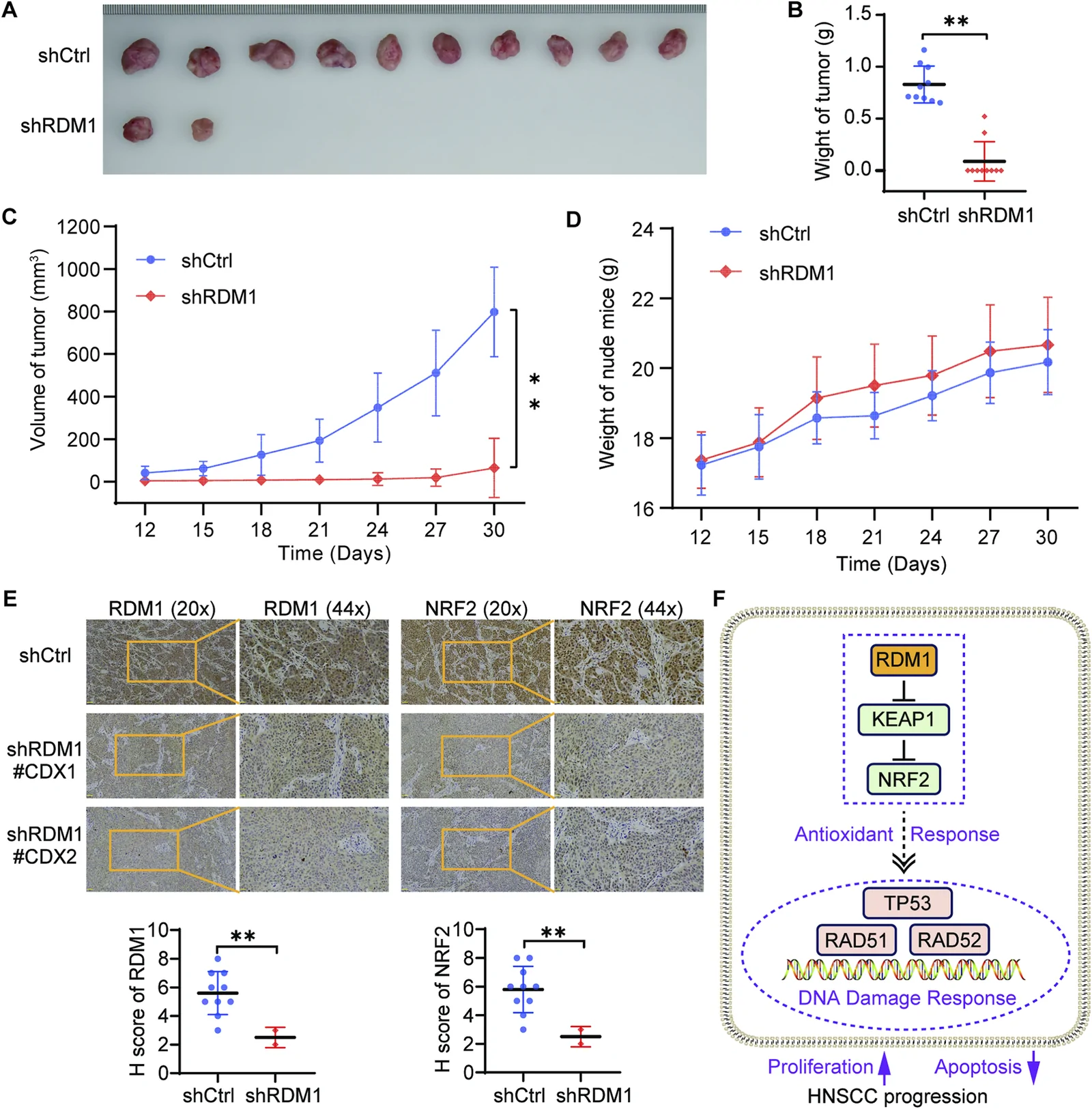
RDM1/NRF2 axis promotes HNSCC progression *in vivo*. **(A**,**B)** Fadu cell-derived xenograft tumors were established in nude mice (n = 10 per group), and the weight of the tumors was measured at the endpoint (day 35). In the shRDM1 group, 2 out of 10 mice developed measurable tumors; non-tumor-bearing animals were assigned a tumor weight of 0. **(C,D)** Growth curves of the xenograft tumors and nude mice were monitored over time (days 12, 15, 18, 21, 24, 27, and 30; tumor growth curve includes only tumor-bearing animals: n = 10 for shCtrl, n = 2 for shRDM1). **(E)** Immunohistochemistry (IHC) was performed to assess the expression levels of RDM1 and NRF2 proteins in the tumor tissues, and statistical analysis was conducted. **(F)** A schematic diagram illustrating the hypothesized role of the RDM1-KEAP1-NRF2 axis in HNSCC progression. Solid arrows indicate experimentally validated relationships; dashed arrow indicates indirectly supported relationships. All data are presented as mean ± SD; *P < 0.05, **P < 0.01. **P value <0.01.

## 4 Discussion

Head and neck squamous cell carcinoma (HNSCC) can be classified into two types based on the presence or absence of the human papillomavirus (HPV). These types differ in their underlying causes of cancer development (Cramer et al., 2019; Jiang et al., 2023). However, both types share a common mechanism of abnormality in DNA damage response (DDR) and genome instability mutation, which promotes cancer progression (Hammerman et al., 2015). This mechanism involves mutations resulting in the loss of CDKN2A and TP53, as well as amplification of CCND1, CDK4, and CDK6 genes. Hence, genomic mutation damage and dysregulated DDR are vital characteristics of HNSCC progression.

RDM1 exhibits a dual role in cancer by both promoting and suppressing its development (Tong et al., 2018; Chen et al., 2019; Tong et al., 2020; Li et al., 2017; Sheng et al., 2021; Xie et al., 2019; Chen et al., 2020). It acts as a promoter in lung cancer, breast cancer, osteosarcoma, and ovarian cancer, whereas it functions as a suppressor in liver cancer. Previous studies have primarily focused on investigating RDM1’s involvement in DDR and cell cycle regulation. Some of these studies have shown that RDM1 increases the levels of DNA repair proteins RAD51 and RAD52 while reducing levels of TP53 and CDKN1A proteins (Tong et al., 2018; Chen et al., 2019; Tong et al., 2020; Sheng et al., 2021). It should be noted that these proteins mainly function in the nucleus, whereas RDM1 is predominantly located in the cytoplasm (Messaoudi et al., 2007). This suggests that RDM1 exerts its function in the cytoplasm and subsequently communicates signals to the nucleus. However, the exact function and molecular mechanism of RDM1 in the cytoplasm remain unclear.

In the current study, we observed that RDM1 exhibited high expression levels in HNSCC tissues (Figure 1). Furthermore, our findings indicated that RDM1 actively contributed to HNSCC progression by promoting cell proliferation while inhibiting apoptosis (Figure 2). Mechanistically, our investigations revealed that RDM1 modulated DDR-associated signaling and cell cycle control by modulating the protein levels of RAD52, TP53, CDKN1A, and CDK6 (Figure 3). This pattern of regulation was consistent with what has been observed in other cancers. RAD52, which is a crucial protein involved in DNA repair and DDR (Bell and Kowalczykowski, 2016), displayed reduced expression levels in cases of abnormal DDR, leading to enhanced TP53 function. Through transcriptional regulation, TP53 influenced various proteins such as CDKN1A, consequently blocking the cell cycle via the inhibitory effect of CDK6 on DNA synthesis (Hernández Borrero and El-Deiry, 2021). Collectively, our findings suggest that RDM1 promotes HNSCC progression by regulating the RAD52/TP53 and CDKN1A/CDK6 axes, thus enhancing both DDR and cell cycle control.

To investigate the molecular mechanism of RDM1 in the cytoplasm, we conducted KEGG enrichment pathway analysis on differentially expressed genes using transcriptome datasets from RDM1 knockdown cells. The analysis revealed that the top pathways affected by RDM1 were MAPK and protein processing in the endoplasmic reticulum (ER), suggesting their involvement in the cytoplasmic function of RDM1. Subsequent experiments confirmed that RDM1 knockdown induced ER stress and elevated levels of reactive oxygen species (ROS) (Figure 4), indicating that RDM1 played a role in the antioxidant response in HNSCC. Additionally, we examined the expression of NRF2 and the effector BCL2 and observed a significant decrease in their protein levels following RDM1 knockdown. Moreover, rescue experiments using NRF2 and BCL2 function demonstrated that RDM1 functioned through the NRF2 signaling pathway (Figure 4). Collectively, these findings provide insights into the molecular mechanism of RDM1 in the cytoplasm.

To investigate how RDM1 enhances NRF2 protein levels, we conducted a screen to identify proteins that interact with RDM1. Our results revealed that RDM1 interacts with KEAP1, an E3 ligase that normally binds to NRF2, leading to its degradation (Rojo de la Vega et al., 2018; Menegon et al., 2016). Based on this finding, we hypothesized that RDM1 competes with NRF2 for binding to KEAP1, thereby preventing NRF2 degradation and enhancing NRF2 signaling. To test this hypothesis, we performed a western blot assay, which showed that RDM1 does not affect the levels of KEAP1 protein. However, overexpression of NRF2 in RDM1 knockdown cells rescued the protein levels of RDM1 (Figure 5). These findings are consistent with a model in which RDM1 binds KEAP1 and interferes with KEAP1-mediated NRF2 degradation, consistent with the canonical KEAP1-mediated ubiquitin–proteasome degradation pathway whereby RDM1 binding may prevent NRF2 ubiquitination and degradation. However, direct competitive binding and protein-stability assays (e.g., competitive Co-IP, cycloheximide-chase, and NRF2 ubiquitination assays) will be necessary to confirm the precise molecular mechanism by which RDM1 stabilizes NRF2. Furthermore, in CDX experiments, we found that RDM1 not only promotes the progression of HNSCC but also plays a role in the regulation of NRF2 (Figure 6). Collectively, our study highlights the critical role of the RDM1–KEAP1–NRF2 axis in HNSCC progression. While our data demonstrate RDM1-dependent regulation of NRF2 protein abundance and associated antioxidant phenotypes, direct assessment of NRF2 transcriptional activity using nuclear accumulation assays, ARE-dependent reporter assays, and canonical target gene validation will be needed to establish the full scope of NRF2 activation. Furthermore, an *in vivo* NRF2 rescue experiment will be necessary to definitively establish that the tumor-suppressive effect of RDM1 knockdown is mediated through NRF2.

Importantly, the NRF2-mediated antioxidant pathway and the DDR machinery are not independent. Three levels of crosstalk connect them: first, NRF2-mediated ROS suppression indirectly reduces oxidative DNA damage and DDR checkpoint activation (Jiang et al., 2025); second, NRF2 may transcriptionally regulate DNA repair genes such as RAD51, consistent with our transcriptomic data showing decreased RAD52 mRNA upon RDM1 knockdown (Yang et al., 2021); third, the NRF2 target BCL2 modulates both apoptosis and cell-cycle progression (Panda et al., 2025). This integrated model suggests that RDM1, by stabilizing NRF2, may simultaneously influence antioxidant defense, DNA repair capacity, and cell-cycle progression in HNSCC.

From a translational perspective, the RDM1–KEAP1–NRF2 axis has therapeutic implications. NRF2 hyperactivation is known to drive cisplatin resistance and radioresistance in HNSCC, and RDM1, acting upstream of NRF2 via KEAP1 interaction, represents a viable target to suppress NRF2 signaling and sensitize tumors to chemo/radiotherapy. RDM1’s high expression in tumors but relatively low expression in most normal tissues suggests a favorable therapeutic window. Future preclinical studies combining RDM1 inhibition with standard-of-care therapy, and development of RDM1–KEAP1 disruptors, are warranted.

Additionally, while our data demonstrate RDM1-dependent regulation of DDR-associated proteins and cell-cycle distribution, direct assessment of DNA damage and repair activity using γH2AX immunostaining, comet assays, or RAD52 foci formation will be needed to establish whether RDM1 directly modulates DNA repair efficiency. Given the established role of DDR in therapeutic response, investigating whether RDM1 modulates sensitivity to cisplatin or radiotherapy represents an important direction for future research.

Several limitations of this study should be acknowledged. The Kaplan-Meier analysis showed only a non-significant trend toward poorer overall survival in the high-RDM1 group, which may reflect TCGA cohort heterogeneity (multiple anatomical subsites, HPV status, and treatment variation) and limited sample size. Therefore, RDM1 should be regarded as a candidate rather than a definitive prognostic indicator at this stage, and future prospective studies in larger, well-characterized cohorts will be needed to properly evaluate its prognostic value.

In conclusion, our study demonstrates that RDM1 plays an oncogenic role in HNSCC, as it is highly expressed in tumor cells and promotes cell proliferation while inhibiting apoptosis. We found that RDM1 modulates DDR-associated signaling and cell cycle through RAD52/TP53 and CDKN1A/CDK6 signaling in HNSCC. Additionally, we investigated the function of RDM1 in the cytoplasm and discovered a new mechanism involving antioxidant response. In this mechanism, RDM1 binds KEAP1 and is associated with enhanced NRF2 protein accumulation (Figure 6F), although the precise competitive mechanism warrants further validation. Our findings reveal the involvement of the RDM1/KEAP1/NRF2 axis in antioxidant response, highlighting potential therapeutic targets for HNSCC treatment. While RDM1 shows promise as a therapeutic target, its prognostic value warrants further validation in larger independent cohorts.

## Data Availability

The data presented in the study have been deposited in the Gene Expression Omnibus (GEO) repository, available at: https://www.ncbi.nlm.nih.gov/geo/query/acc.cgi?acc=GSE348811.
