# Supplementary material for: Unraveling the role of RDM1 in head and neck squamous cell carcinoma: insights into DDR regulation and antioxidant response
Source: Front Mol Biosci. 2026 Oct 13;13:1944888. doi: 10.3389/fmolb.2026.1944888 (PMC13710011; doi:10.3389/fmolb.2026.1944888)
Supplement: Supplementary file 1 [file DataSheet1.docx]

**Supplementary data**

**Unraveling the Role of RDM1 in Head and Neck Squamous Cell Carcinoma: Insights into DDR Regulation and Antioxidant Response**

**Table S1**

**Table S1.** RDM1 mRNA expression levels were assessed in the HNSCC cohort*

| **Feature** | **log2****FoldChange** | **pValue** | **FDR** |
| --- | --- | --- | --- |
| RDM1 | 1.21 | 4.34E-10 | 1.30E-08 |

*100 tumor and normal tissues. Reference: Huang C, Chen L, Savage S, Eguez R, Dou Y, Li Y, da Veiga Leprevost F, Jaehnig E, Lei J, Wen B, *et al*: Proteogenomic insights into the biology and treatment of HPV-negative head and neck squamous cell carcinoma. Cancer cell 2021, 39:361-379.e316. (supplemental information table S1 sheet “RNA_differential_TumorNormal”)

**Table S2**

**Table S2.** Expression of canonical NRF2 target genes in Transcriptomic Data

| Symbol | si-NC | si-NC | si-NC | si-RDM1 | si-RDM1 | si-RDM1 | FoldChange | pValue |
| --- | --- | --- | --- | --- | --- | --- | --- | --- |
| BRCA1 | 7.08 | 7.16 | 7.11 | 5.82 | 5.86 | 5.82 | -2.44 | 2.3E-12 |
| OGG1 | 6.49 | 6.50 | 6.72 | 5.69 | 5.79 | 5.15 | -2.03 | 6.3E-07 |
| MUTYH | 8.31 | 8.38 | 8.32 | 7.25 | 7.32 | 7.16 | -2.14 | 8E-13 |
| MSH6 | 9.09 | 9.07 | 8.99 | 7.611 | 7.74 | 7.48 | -2.71 | 2.9E-14 |

**Table S3**

**Table S3.** Key candidate interacting proteins of RDM1

| **Protein symbol** | **IP-Mass (Abundances)** | | | **HuProt microarray** |
| --- | --- | --- | --- | --- |
|  | **OE** | **NC** | **FoldChange** | **SNR 635(Mean)** |
| BSG | 20799746 | 138267 | 150.43 | 4.72 |
| NDUFS1 | 10310562 | 106369 | 96.93 | 4.01 |
| SLC25A13 | 29501913 | 434445 | 67.91 | 5.21 |
| IMMT | 23096440 | 570786 | 40.46 | 2.60 |
| THBS1 | 3826716 | 99634 | 38.41 | 3.45 |
| TP63 | 58749037 | 1622920 | 36.20 | 4.92 |
| HSD17B8 | 4222027 | 117427 | 35.95 | 3.42 |
| AIFM1 | 103953058 | 3929390 | 26.46 | 5.15 |
| TFRC | 104759030 | 4715851 | 22.21 | 2.51 |
| NDUFA10 | 8399562 | 444868 | 18.88 | 3.94 |
| TIGAR | 1006903 | 64471 | 15.62 | 6.50 |
| FKBP8 | 7054345 | 482549 | 14.62 | 4.17 |
| FHL2 | 13973901 | 969854 | 14.41 | 3.26 |
| NDUFS2 | 4396982 | 321508 | 13.68 | 2.06 |
| SQSTM1 | 39390289 | 2909673 | 13.54 | 4.22 |
| TRAF2 | 2150365 | 170328 | 12.62 | 3.01 |
| HDAC1 | 33559244 | 2983778 | 11.25 | 3.04 |
| LETM1 | 2374366 | 212218 | 11.19 | 3.40 |
| ATP2A2 | 143524324 | 12939763 | 11.09 | 2.36 |
| DNAJA1 | 91678961 | 8878908 | 10.33 | 8.65 |
| ABCE1 | 5482110 | NA | \ | 5.56 |
| AIFM2 | 236059 | NA | \ | 11.59 |
| BRAT1 | 941415 | NA | \ | 5.26 |
| C1QBP | 497314 | NA | \ | 5.18 |
| CIAO1 | 490638 | NA | \ | 11.57 |
| DGKA | 754898 | NA | \ | 6.34 |
| ERAL1 | 17805109 | NA | \ | 9.38 |
| FOXK2 | 2045451 | NA | \ | 5.12 |
| **KEAP1** | **1800636** | **NA** | **\** | **5.31** |
| MCL1 | 305062 | NA | \ | 5.96 |
| NDUFAF1 | 469846 | NA | \ | 10.55 |
| NDUFB11 | 1061446 | NA | \ | 5.19 |
| NDUFS3 | 2529123 | NA | \ | 5.41 |
| NDUFS8 | 150880 | NA | \ | 5.13 |
| OGDH | 15748335 | NA | \ | 7.35 |
| PELO | 1367364 | NA | \ | 5.60 |
| PLK1 | 2065959 | NA | \ | 6.43 |
| PRPS1 | 11912151 | NA | \ | 7.19 |
| PTPN2 | 1262472 | NA | \ | 14.15 |
| RAB32 | 200946 | NA | \ | 12.07 |
| RAC1 | 3382513 | NA | \ | 7.04 |
| RB1 | 5910434 | NA | \ | 5.44 |
| RHOA | 5373181 | NA | \ | 7.58 |
| SFXN3 | 12075492 | NA | \ | 5.14 |
| SLC25A21 | 6527856 | NA | \ | 7.36 |
| SLC25A36 | 779817 | NA | \ | 5.68 |
| STAT2 | 248400 | NA | \ | 5.48 |
| TIMM44 | 6707667 | NA | \ | 7.00 |
| TIMMDC1 | 755988 | NA | \ | 6.05 |
| TXLNA | 1215616 | NA | \ | 7.14 |
| PUM1 | 13595791 | NA | \ | 6.23 |
| AGK | 8156876 | NA | \ | 2.41 |
| SDHA | 45169927 | NA | \ | 2.83 |
| SLC25A19 | 7993875 | NA | \ | 2.46 |
| TBRG4 | 28460682 | NA | \ | 4.01 |
| TPM1 | 3950868 | NA | \ | 3.23 |
| DDX20 | 38444238 | NA | \ | 3.90 |

**Figure S1**

**Fig. S1.** The relative expression of RDM1 in tumor and normal tissues from patients with HNSCC in TCGA cohort.

**Figure S2**

**Fig. S2.** Highly expressed RDM1 promotes cell proliferation and suppresses apoptosis in HNSCC cells.

**A**. The efficiency of RDM1 knockdown (siRDM1) was confirmed using RT-qPCR. **B**. The cell viability of RDM1 knockdown was evaluated using the CCK-8 assay. **C**. Cell apoptosis in RDM1 knockdown was measured using the Annexin V-APC apoptosis assay.
